# Supplementary material for: CB2 improves power of cell detection in droplet-based single-cell RNA sequencing data
Source: Genome Biol. 2020 Jun 8;21:137. doi: 10.1186/s13059-020-02054-8 (PMC7278076; doi:10.1186/s13059-020-02054-8)
Supplement: Supplementary file 2 — Additional file 2: Table S1. The number of cells identified by CB2, ED, or both in 11 case study datasets. Table S2. Links to all datasets used in this study. Table S3. Number of novel subpopulations identified by CB2 in each dataset. [file 13059_2020_2054_MOESM2_ESM.docx]

**Supplementary Tables**

CB2 improves power of cell detection in droplet-based single-cell RNA sequencing data

Zijian Ni (zni25@wisc.edu)^1^, Shuyang Chen (schen662@wisc.edu)^1^, Jared Brown (brown46@wisc.edu)^1^, Christina Kendziorski (c.kendziorski@gmail.com)^2*^

^1^Department of Statistics, University of Wisconsin-Madison, Madison, WI, USA

^2^Department of Biostatistics and Medical Informatics, University of Wisconsin-Madison, Madison, WI, USA

*Corresponding author

| **Dataset** | **High-count cells**  **(untested)** | **Tested cells identified by both CB2 and ED** | **Cells uniquely identified by CB2** | **Cells uniquely identified by ED** | **CB2 unique cells in existing subpopulation** | **CB2 unique cells in novel subpopulation** |
| --- | --- | --- | --- | --- | --- | --- |
| Alzheimer | 12143 | 57278 | 10689 / 57278  (18.66%) | 50 / 57278  (0.09%) | 6819 / 10689  (63.79%) | 3870 / 10689  (36.21%) |
| PBMC8K | 6708 | 1445 | 1165 / 1445  (80.62%) | 2 / 1445  (0.14%) | 1165 / 1165  (100%) | 0 / 1165  (0%) |
| PBMC33K | 23491 | 11762 | 424 / 11762  (3.60%) | 0 / 11762  (0%) | 424 / 424  (100%) | 0 / 424  (0%) |
| mbrain1K | 581 | 1469 | 221 / 1469  (15.04%) | 16 / 1469  (1.09%) | 166 / 221  (75.11%) | 55 / 221  (24.89%) |
| mbrain9K | 6048 | 5685 | 1265 / 5685  (22.25%) | 98 / 5685  (1.72%) | 1057 / 1265  (83.56%) | 208 / 1265  (16.44%) |
| PanT4K | 3398 | 1700 | 261 / 1700  (15.35%) | 0 / 1700  (0%) | 261/ 261  (100%) | 0 / 261  (0%) |
| MALT | 3378 | 981 | 494 / 981  (50.36%) | 2 / 981  (0.20%) | 216 / 494  (43.72%) | 278 / 494  (56.28%) |
| PBMC4K | 2145 | 6516 | 1003 / 6516  (15.39%) | 0 / 6516  (0%) | 1003 / 1003  (100%) | 0 / 1003  (0%) |
| jurkat | 2565 | 953 | 175 / 953  (18.36%) | 0 / 953  (0%) | 175 / 175  (100%) | 0 / 175  (0%) |
| T293 | 2299 | 797 | 48 / 797  (6.02%) | 2 / 797  (0.25%) | 48 / 48  (100%) | 0 / 48  (0%) |
| placenta | 4349 | 2947 | 637 / 2947  (21.62%) | 1 / 2947  (0.03%) | 637 / 637  (100%) | 0 / 637  (0%) |

**Table S1:** The number of cells identified by CB2, ED, or both in 11 case study datasets.

| **Dataset** | **Link** |
| --- | --- |
| Alzheimer | <https://www.synapse.org/#!Synapse:syn16780177> |
| PBMC8K | <https://support.10xgenomics.com/single-cell-gene-expression/datasets/2.1.0/pbmc8k> |
| PBMC33K | <https://support.10xgenomics.com/single-cell-gene-expression/datasets/1.1.0/pbmc33k> |
| mbrain1K | <https://support.10xgenomics.com/single-cell-gene-expression/datasets/2.1.0/neurons_900> |
| mbrain9K | <https://support.10xgenomics.com/single-cell-gene-expression/datasets/2.1.0/neuron_9k> |
| PanT4K | <https://support.10xgenomics.com/single-cell-gene-expression/datasets/2.1.0/t_4k> |
| MALT | <https://support.10xgenomics.com/single-cell-gene-expression/datasets/3.0.0/malt_10k_protein_v3> |
| PBMC4K | <https://support.10xgenomics.com/single-cell-gene-expression/datasets/2.1.0/pbmc4k> |
| jurkat | <https://support.10xgenomics.com/single-cell-gene-expression/datasets/1.1.0/jurkat> |
| T293 | <https://support.10xgenomics.com/single-cell-gene-expression/datasets/1.1.0/293t> |
| placenta | <https://jmlab-gitlab.cruk.cam.ac.uk/publications/EmptyDrops2017-DataFiles> |

**Table S2**: Links to all datasets used in this study.

| **Dataset** | **Threshold** | | |
| --- | --- | --- | --- |
|  | **90%** | **80%** | **70%** |
| Alzheimer | 1 | 2 | 2 |
| PBMC8K | 0 | 0 | 0 |
| PBMC33K | 0 | 0 | 0 |
| mbrain1K | 0 | 1 | 1 |
| mbrain9K | 0 | 1 | 1 |
| PanT4K | 0 | 0 | 0 |
| MALT | 1 | 1 | 2 |
| PBMC4K | 0 | 0 | 0 |
| jurkat | 0 | 0 | 1 |
| T293 | 0 | 0 | 0 |
| placenta | 0 | 0 | 1 |

**Table S3**: Number of novel subpopulations identified by CB2 in each dataset.
