# Supplementary material for: CB2 improves power of cell detection in droplet-based single-cell RNA sequencing data
Source: Genome Biol. 2020 Jun 8;21:137. doi: 10.1186/s13059-020-02054-8 (PMC7278076; doi:10.1186/s13059-020-02054-8)
Supplement: Supplementary file 3 — Additional file 3. Review history. [file 13059_2020_2054_MOESM3_ESM.docx]

Review History

**First round of review**

**Reviewer 1**

**Were you able to assess all statistics in the manuscript, including the appropriateness of statistical tests used?**

Yes, and I have assessed the statistics in my report.

**Comments to author:**

Ni et al. describe in their manuscript a bioinformatic method to increase the number of cells identified in droplet single cell RNA-sequencing (scRNA-seq) datasets. In droplet scRNA-seq experiments, mRNA capture happens on barcoded beads and the gene expression matrix is constructed by grouping together sequencing reads by barcode. To avoid double encapsulations cells are typically loaded at a low concentration such that only about 10% of droplets contain a cell; this factor and molecular biology and sequencing errors lead to an output gene expression table where there are orders of magnitude more barcodes than are expected cells. An ongoing challenge in the field is to determine which of these barcodes correspond to actual cells, and the authors describe in the introduction various methods, with different levels of sophistication, to tackling this challenge.

Ultimately the authors make a direct comparison to a previously published cell calling method, EmptyDrops (ED). Briefly, ED divides barcodes into three main groups: those that are highly likely to be cells, those that are highly unlikely to be cells, and those in the middle. It tests every barcode in the middle group to a background signal obtained by averaging those barcodes that are highly unlikely to be cells, and those barcodes that are significantly different it classifies as cells. The author's method, termed CB2, expands on this approach by first clustering barcodes in the middle group and then assessing whether those clusters significantly differ from the background. Thus in CB2, entire groups of barcodes may at once be assigned to be cells rather than on a case-by-case basis as with ED. The authors test CB2 on an existing scRNA-seq datasets, demonstrating that they are able to identify additional cells compared with ED, and that these additional cells cluster with existing clusters or form new clusters.

I believe that there is merit in CB2 to improving the analysis of gene expression matrices obtained with droplet scRNA-seq workflows, though I have a few concerns that could affect the utility of the method that the authors have not explicitly addressed within the manuscript:

1. Where in the distribution of UMI counts vs barcodes do the additional cells identified by CB2 lie? Could a majority of the additional cells be obtained by lowering the 'upper threshold' as defined in Fig S2? If so this would obviate the need for the increased processing cost of implementing CB2.

2. If cells were randomly sampled from the area skirting the 'lower threshold' line as defined in Fig S2, where would they cluster on the tSNE? Would cells from the background barcodes also display similar clustering patterns to the additional cells identified through CB2?

3. The unique subpopulation (Subpop 5) identified in Figure 2 is one of the more compelling results of the manuscript, yet glossed over within the rest of the figure. The authors claim that these cells express a mixture of genes from two other clusters, which could also just be the result of beads capturing ambient RNA within the sample or cell fragments. More rigorous analysis and confirmation of new clusters obtained through this method should be done.

4. The application of CB2 seems to introduce many additional cells within Subpop2 in Figure 2. In addition, these cells are not well mixed with the other cells in the cluster that were identified with the standard workflow. What is this significance that these cells cluster differently from the rest of the data?

Overall I believe that if the results are to be taken at face value CB2 is an advance over the existing approaches for analyzing droplet scRNA-seq data, though I would like the authors to provide more conclusive backing that these results are from cells and not cell fragments or background RNA.

**Reviewer 2**

**Were you able to assess all statistics in the manuscript, including the appropriateness of statistical tests used?**

Yes, and I have assessed the statistics in my report.

**Comments to author:**

This manuscript presents CB2, a method for detecting cell-enclosing droplets in scRNA-seq. The paper tries to solve the problem where in scRNA-seq, cell-enclosing droplets are intermixed with empty droplets, which contain ambient RNA molecules that drift in the cell assay. Traditionally, analytics sets a UMI threshold and all droplets whose total UMI counts are smaller than the threshold are classified as empty droplets and are subsequently removed. The authors argue

that some cell types might naturally produce small-UMI-count droplets, due to having smaller sizes. Crude classification methods such as using a single UMI count threshold could incorrectly exclude all droplets of an entire cell type from the dataset.

Currently, the state-of-the-art cell detection method, ED, uses a more refined three-step classification approach. First, ED uses two UMI count thresholds to categorize droplets: a high-UMI-count droplet group, a low-UMI-count droplet group, and the third group where droplets have intermediate UMI counts between the two thresholds. The low-UMI-count droplet group are assumed to be empty droplets. Second, ED fits a Dirichlet-Multinomial model to the low-UMI-count empty droplet group, in order to characterize the ambient RNA distribution in empty droplets. Finally, it tests every droplet in the intermediate-UMI-count droplet group and check if it is statistically significant that the droplet is empty.

CB2 further extends the frame work of ED. CB2 clusters intermediate-UMI-count droplets prior to significance testing and tests if the entire cluster is made up of empty droplets, instead of testing droplets individually. The main idea is that when tested individually, there might not be sufficient statistical power to reject the assumption that a cell-enclosing droplet is not empty. When tested collectively as a cluster, if multiple cells have mutually similar expression profiles that are different from the background noise, there will be more statistical powers to reject the assumption. The authors benchmarked CB2 against ED on ten real-data-driven simulated scRNA-seq datasets, as well as three real datasets. The results prove that CB2 is able to rescue more cells and preserve rare cell types in scRNA-seq datasets. The structure of the paper is clear, although the clarity of the writing can be improved. The paper is technically sound. However, we believe that the quality of the paper can be further improved after addressing the following comments:

* The paper assumes that there exists cell types whose UMI counts are consistently lower than other cell types. This assumption was not clearly conveyed, nor was it extensively validated. Figure 2 somewhat shows evidence that supports the idea (with the Subpop5 cluster) but was not further discussed. In Figure 2d, none of the marker genes have high expression values in Subpop5. It is not clear if Subpop5 are real cells of a certain type. Instead, the paper emphasizes CB2's ability to expand the Subpop2 cluster, which originally has half of the population included by ED.

* It is also not clear how are CB2-unique droplets different from the common droplets in Subpop2. Perhaps the authors can annotate the Subpop5 cluster with a cell type and prove that it is not just background noise; and show the overall UMI count distribution across all droplets.

* Just like ED, CB2 also uses two sets of threshold for the initial 3-group classification. It is evident that these two thresholds have an impact over the performance of both ED and CB2. Similarly, the naive approach (single threshold) also uses thresholds. The paper did not provide sufficient evidence to support its intuition where there exists cell-enclosing droplets whose overall UMI counts are similar to empty droplets. Perhaps the naive approach has worse performance simply due to using non-optimal thresholds. We recommend the authors to provide further evidence to show cell-enclosing droplets could indeed have similar UMI counts to empty droplets. A evaluation over the impact of parameter choices between naive, ED and CB2 is also much desired.

* The rationale behind the simulation setup is not well explained in the manuscript. The authors simulate datasets by mixing the original dataset with multiple down-sized versions of itself at different scaling factors. This comment is also attached to the previous two comments. The simulation approach suggests that all cell types have equal chances of being under sampled in sequencing and become indistinguishable from the background noise. Without providing justifications that there does not exist a clear cut between cell-enclosing droplets and empty droplets in terms of total UMI counts and all cell types may get down sampled, it renders the simulation setup somewhat random and arbitrary.

* The significance of conducting statistical tests over an entire cluster instead of individual droplets is not clearly explained and, in our opinion, not sufficiently stressed.

Minor comments:

* Page 3 line 31-43. ED is poorly explained. "Similar to previous approaches" -> add citation. Does ED infers the upper and the lower threshold from the dataset? "compromises power in many datasets" -> unclear. Perhaps "compromises its statistical power in identifying cells in many datasets"?

*Page 4 line 19-29: hard to follow. Adding an overview of the simulation process would greatly help. Such as: simulation is conducted by combining three differently scaled datasets G1, G2 and G3.

*Page 4 line 48: "To further evaluate CB2, we applied CB2 and ED to the ten datasets evaluated in the simulation study". Is it not simulation? Is it a repeat of the previous paragraph or new results? Unclear.

*The github has very limited information and example. A clear documentation with running examples should be provided so readers can follow and apply to their own datasets.

**Authors Response**

**Point-by-point responses to the reviewers’ comments:**

Reviewer #1

Ni et al. describe in their manuscript a bioinformatic method to increase the number of cells identified in droplet single cell RNA-sequencing (scRNA-seq) datasets. In droplet scRNA-seq experiments, mRNA capture happens on barcoded beads and the gene expression matrix is constructed by grouping together sequencing reads by barcode. To avoid double encapsulations cells are typically loaded at a low concentration such that only about 10% of droplets contain a cell; this factor and molecular biology and sequencing errors lead to an output gene expression table where there are orders of magnitude more barcodes than are expected cells. An ongoing challenge in the field is to determine which of these barcodes correspond to actual cells, and the authors describe in the introduction various methods, with different levels of sophistication, to tackling this challenge.

Ultimately the authors make a direct comparison to a previously published cell calling method, EmptyDrops (ED). Briefly, ED divides barcodes into three main groups: those that are highly likely to be cells, those that are highly unlikely to be cells, and those in the middle. It tests every barcode in the middle group to a background signal obtained by averaging those barcodes that are highly unlikely to be cells, and those barcodes that are significantly different it classifies as cells. The author's method, termed CB2, expands on this approach by first clustering barcodes in the middle group and then assessing whether those clusters significantly differ from the background. Thus in CB2, entire groups of barcodes may at once be assigned to be cells rather than on a case-by-case basis as with ED. The authors test CB2 on an existing scRNA-seq datasets, demonstrating that they are able to identify additional cells compared with ED, and that these additional cells cluster with existing clusters or form new clusters.

I believe that there is merit in CB2 to improving the analysis of gene expression matrices obtained with droplet scRNA-seq workflows, though I have a few concerns that could affect the utility of the method that the authors have not explicitly addressed within the manuscript:

1. Where in the distribution of UMI counts vs barcodes do the additional cells identified by CB2 lie?

*Below we show the distribution of UMI counts for background barcodes and cells identified uniquely by CB2 (left panel), identified in total by CB2 (middle panel), and identified by ED (right panel) for the Alzheimer dataset. As shown, the additional cells identified by CB2 are in the moderate count range [the 25th and 75th percentile of UMI counts for barcodes that are not background by default is 423-3873].*

*Figure: Distribution of the number of cells and background barcodes identified uniquely by CB2 (left), identified in total by CB2 (middle), and identified by ED (right). The x-axis is log10 scaled ranging from lower threshold (100) to upper threshold (for the Alzheimer dataset, the upper threshold is 8136) since barcodes outside this range are not tested by either CB2 or ED.*

Could a majority of the additional cells be obtained by lowering the 'upper threshold' as defined in Fig S2? If so this would obviate the need for the increased processing cost of implementing CB2.

*Yes, if a simple threshold existed it would obviate the need for CB2 (and ED). However, a simple thresholding method does not work in practice. An example of this is given in the figure above where there is no single threshold that separates background barcodes from real cells.*

*Having said that, we acknowledge that the figure above shows data from a single case study where the truth is not known, and so we address your question in more detail using data from SIMIA. In particular, for SIMIA datasets we evaluated two simple thresholding approaches: T1 refers to identifying a barcode as a cell if the total count exceeds 50% of the inflection point and T2 is similar, but at 90% of the inflection point. As shown below, the T1 threshold does increase power in 7 of the 10 simulated datasets considered. However, in most of these datasets the increased power comes with greatly increased false discovery rates. In particular, at the T1 threshold, 9 of the 10 simulated datasets considered have FDR above the 1% target; 3 of the 10 have FDRs over 20%. Using the T2 threshold only increases power in 4 of the datasets and for 3 of these, the increase is less than 2%. However, as with the T1 threshold, the increased power comes with increased FDR. For T2, 3 of the 10 datasets have FDR that is not well controlled at the target 1%.*

*Figure: Average power (top panel) and average FDR (lower panel) from two thresholding methods (T1 and T2) and CB2 applied to SIMIA data (power and FDR are averaged over G1, G1.5, and G2 cells). In T1, barcodes are classified as cells if their total UMI count exceeds 50% of the inflection point. T2 is similar, but at 90% of the inflection point.*

2. If cells were randomly sampled from the area skirting the 'lower threshold' line as defined in Fig S2, where would they cluster on the tSNE?

*Cells identified by CB2 that are “skirting the lower threshold” form clusters separate from the background, as shown below. Specifically, by “skirting the lower threshold”, we took cells identified by CB2 with UMI counts in the range 101 to 200. For comparison, we also added barcodes that are called background automatically (those with UMI counts less than 100) and those called background after testing by CB2 having counts between 101 to 200. These groups tend to form separate clusters.*

*Figure: t-SNE plot from the Alzheimer’s dataset showing CB2 cells with UMI counts in the range 101-200 as well as background barcodes with counts less than 100 and with counts in the range 101-200. Because there are over 6 million barcodes with counts less than 100, we sampled 2000 barcodes within each background group for visualization.*

Would cells from the background barcodes also display similar clustering patterns to the additional cells identified through CB2?

*By “additional cells”, we assume you mean the cells identified by CB2 but not ED. As shown above for the cells identified by CB2 that are relatively low count, cells identified uniquely by CB2 also cluster distinctly from the background barcodes. To illustrate this, the figure below (left panel) shows results from the Alzheimer’s dataset with background barcodes separated into two groups (those with counts less than 100 and those with counts greater than 100). The background barcodes are largely distinct from the unique CB2 identifications. Results from the PBMC8K are similar (shown in the right panel).*

*Figure: t-SNE plot from the Alzheimer’s (left) and PBMC8K (right) datasets showing cells identified uniquely by CB2 as well as background barcodes separated into two groups (those with counts less than 100 and those with counts greater than 100). Because there are over 6 million barcodes with counts less than 100, we sampled 2000 barcodes within each background group for visualization.*

3. The unique subpopulation (Subpop 5) identified in Figure 2 is one of the more compelling results of the manuscript, yet glossed over within the rest of the figure. The authors claim that these cells express a mixture of genes from two other clusters, which could also just be the result of beads capturing ambient RNA within the sample or cell fragments. More rigorous analysis and confirmation of new clusters obtained through this method should be done.

*Thank you. We regret that we did not focus on Subpop5 in sufficient detail. Given the assumed mixing of ambient mRNA in the microfluidics based 10x technology, it is unlikely that we would observe barcodes binding ambient mRNA largely from two specific subpopulations, but not others. Specifically, suppose there are 5 subpopulations in the sample with ambient RNA fragments in proportions 40%, 40%, 10%, 5%, and 5%. The probability that we would see an ambient barcode (barcode binding ambient RNA) with total UMI count 100 having 0% fragments in the last three subpopulations is*

*0.8$°° = 2.04 × 10-$°*

*Observing a barcode with total UMI count 100 having less than 5% fragments in the last three subpopulations is*

*4*

*,-100 / 0.21 × 0.8$°°-1 1=°	= 3.70 × 10-6*

*It is important to note, however, that if/when these unlikely events happen, CB2 (and ED for that matter) will mistakenly identify an ambient barcode as a real cell since its expression distribution differs from the background overall. Take for example low quality (or dying) cells showing high expression of mitochondrial genes. Both CB2 and ED will identify barcodes tagging mRNA from these cells as real cells given that they show expression patterns that differ from the background. In the ED paper, the authors note “a notable side-effect of retaining barcodes with low UMI totals is that a higher number of low-quality cells are also recovered. EmptyDrops is technically correct in retaining the associated barcodes as damaged cells are distinct from empty droplets. However, these cells are usually not of interest in downstream analyses. We have removed them by thresholding on the mitochondrial content... If this is not sufficient, manual inspection of the clustering results may be necessary to identify these cells and exclude them from further consideration.” We have provided a filter in the R/CB2 package for a similar reason.*

*In an effort to further convince a reader that the barcodes identified in Subpop5 appear to be different from the background, as with other subpopulations we now include distribution plots of the 100 genes having highest average expression in Subpop5. We have added these plots to Supplementary Figure 6 (they are shown below for convenience). As shown, cells uniquely identified by CB2 in Subpop5 have a distribution similar to Subpop5 cells identified by ED and they differ from the background.*

*Figure: Distribution plots of the 100 genes having highest average expression in Subpop5 are shown for cells identified by both CB2 and ED (upper) and identified uniquely by CB2 (middle). The estimated background distribution is also shown (lower). Cells uniquely identified by CB2 in Subpop5 have a distribution similar to other Subpop5 cells and differ from the background.*

4. The application of CB2 seems to introduce many additional cells within Subpop2 in Figure 2. In addition, these cells are not well mixed with the other cells in the cluster that were identified with the standard workflow. What is this significance that these cells cluster differently from the rest of the data?

*Great question. By testing on clusters of barcodes, CB2 improves the power to identify cells having relatively low UMI counts and as a result many of the cells identified uniquely by CB2 have lower counts than those identified in common by both CB2 and ED. Given that t-SNE plots are sensitive to total counts (Townes et al., 2019), we often see the CB2 unique identifications less well mixed with the other identifications. Below we show a t-SNE plot where barcodes are downsampled so that all identifications have the same total UMI count. After removing the effect of total counts, CB2 unique cells are well-mixed with the cells identified in common by both CB2 and ED.*

*Figure: t-SNE plot for Subpop2 (from Figure 2 in the manuscript) before (left) and after (right) down-sampling. After down-sampling, the effect of total counts is removed, and CB2 cells are well-mixed with common cells.*

Overall I believe that if the results are to be taken at face value CB2 is an advance over the existing approaches for analyzing droplet scRNA-seq data, though I would like the authors to provide more conclusive backing that these results are from cells and not cell fragments or background RNA.

*Thank you. We hope that our additional analyses have provided sufficient support for CB2.*

Reviewer #2

This manuscript presents CB2, a method for detecting cell-enclosing droplets in scRNA-seq. The paper tries to solve the problem where in scRNA-seq, cell-enclosing droplets are intermixed with empty droplets, which contain ambient RNA molecules that drift in the cell assay. Traditionally, analytics sets a UMI threshold and all droplets whose total UMI counts are smaller than the threshold are classified as empty droplets and are subsequently removed. The authors argue that some cell types might naturally produce small-UMI-count droplets, due to having smaller sizes. Crude classification methods such as using a single UMI count threshold could incorrectly exclude all droplets of an entire cell type from the dataset.

Currently, the state-of-the-art cell detection method, ED, uses a more refined three-step classification approach. First, ED uses two UMI count thresholds to categorize droplets: a high-UMI-count droplet group, a low-UMI-count droplet group, and the third group where droplets have intermediate UMI counts between the two thresholds. The low-UMI-count droplet group are assumed to be empty droplets. Second, ED fits a Dirichlet-Multinomial model to the low-UMI-count empty droplet group, in order to characterize the ambient RNA distribution in empty droplets. Finally, it tests every droplet in the intermediate-UMI-count droplet group and check if it is statistically significant that the droplet is empty.

CB2 further extends the frame work of ED. CB2 clusters intermediate-UMI-count droplets prior to significance testing and tests if the entire cluster is made up of empty droplets, instead of testing droplets individually. The main idea is that when tested individually, there might not be sufficient statistical power to reject the assumption that a cell-enclosing droplet is not empty. When tested collectively as a cluster, if multiple cells have mutually similar expression profiles that are different from the background noise, there will be more statistical powers to reject the assumption. The authors benchmarked CB2 against ED on ten real-data-driven simulated scRNA-seq datasets, as well as three real datasets. The results prove that CB2 is able to rescue more cells and preserve rare cell types in scRNA-seq datasets. The structure of the paper is clear, although the clarity of the writing can be improved. The paper is technically sound. However, we believe that the quality of the paper can be further improved after addressing the following comments:

The paper assumes that there exists cell types whose UMI counts are consistently lower than other cell types. This assumption was not clearly conveyed, nor was it extensively validated.

*We regret that this was not clear. CB2 does not assume that there exist cell types with UMI counts that are consistently lower than other cell types. By testing on clusters of barcodes, CB2 does improve the power to identify cells having relatively low UMI counts, but the vast majority of cells uniquely called by CB2 are within cell types that are also identified by ED, for example. CB2 simply finds additional cells within those cell types (as shown in subpopulations 1-4 of Figure 2).*

*There are some exceptions to this. For example, CB2 identified a novel subpopulation of cells, Subpop5 (also shown in Figure 2), but the subpopulation does not have counts that are uniformly lower than other subpopulations. This is discussed in more detail in response to your next question.*

Figure 2 somewhat shows evidence that supports the idea (with the Subpop5 cluster) but was not further discussed.

*We are not sure what you mean here, but are assuming that you are asking whether Subpop5 has counts that are uniformly lower than other subpopulations. As shown in the plot below, it does not.*

*Figure: Boxplot of total UMI counts of cells identified uniquely by CB2 and cells identified by both CB2 and ED for five subpopulations found in the Alzheimer’s data.*

In Figure 2d, none of the marker genes have high expression values in Subpop5. It is not clear if Subpop5 are real cells of a certain type. Instead, the paper emphasizes CB2's ability to expand the Subpop2 cluster, which originally has half of the population included by ED.

*Yes, none of the marker genes in Figure 2d show high expression for Subpop5 since they are neuronal markers and Subpop5 cells appear to be glial (non-neuronal) cells. As mentioned, Subpop5 cells show high expression of oligodendrocyte and astrocyte marker genes.*

*As for whether or not Subpop5 is made up of real cells (defined as cells that differ from the background), we regret that we did not provide more evidence to convince a reader. With other subpopulations, we had presented a distribution of expression for the top 100 genes. We have now added that plot to Supplementary Figure 6; it is shown below for convenience. As shown, cells uniquely identified by CB2 in Subpop5 have a distribution similar to Subpop5 cells identified by ED and they differ from the background.*

*Figure: Distribution plots of the 100 genes having highest average expression in Subpop5 are shown for cells identified by both CB2 and ED (upper) and identified uniquely by CB2 (middle). The estimated background distribution is also shown (lower). Cells uniquely identified by CB2 in Subpop5 have a distribution similar to other Subpop5 cells and differ from the background.*

It is also not clear how are CB2-unique droplets different from the common droplets in Subpop2.

*Reviewer #1 asked a similar question, and we reiterate our response here. We acknowledge that the cells uniquely identified by CB2 in Subpop2 appear to be shifted in the t-SNE plot relative to the commonly identified cells. This is because t-SNE plots are sensitive to total counts (Townes et al., 2019). Because of this, we often see the CB2 unique identifications less well mixed with the other identifications. Below we show a t-SNE plot where barcodes are downsampled so that all identifications have the same total UMI count. After removing the effect of total counts, CB2 unique cells are well-mixed with the cells identified in common by both CB2 and ED.*

*Figure: t-SNE plot for Subpop2 (from Figure 2 in the manuscript) before (left) and after (right) down-sampling. After down-sampling, the effect of total counts is removed, and CB2 cells are well-mixed with common cells.*

Perhaps the authors can annotate the Subpop5 cluster with a cell type and prove that it is not just background noise; and show the overall UMI count distribution across all droplets.

*As for annotating Subpop5, we considered all markers used in Mathys et al., Nature, 2019 for identifying cell type. Following their approach for classifying cells, cells in Subpop5 would be considered both oligodendrocyte and astrocyte cells as they show relatively high expression of GFAP (astrocyte marker) and MBP (oligodendrocyte marker). These types of cells (showing high expression of these two markers) are also known as mixed phenotype glial cells, as noted. Below we show expression of these markers for Subpop5 cells and Subpop4 cells, for comparison. As shown Subpop4 cells show relatively low expression of these markers, but high expression of GAD1 and GAD2 which are markers for inhibitory neuronal cells.*

*Figure: Distribution plots of GAD1 and GAD2 (inhibitory neuronal marker genes) as well as GFAP (astrocyte marker) and MBP (oligodendrocyte marker) for Subpop4 cells (characterized as neuronal cells) and Subpop5 cells (characterized as mixed phenotype glial cells as they show relatively high expression of both astrocyte and oligodendrocyte markers).*

*As for the overall UMI count distribution in Subpop5, it is shown below. If you were asking about the UMI count distribution across all barcodes (not just those from Subpop5), it is shown below after the next response.*

*Figure: Distribution of the number of cells identified by CB2 for Subpop5 and all background barcodes. The x-axis is log10 scaled ranging from lower threshold (100) to the total count of the largest cell in Subpop5 (15579). The y-axis is also log10 scaled since there are much more background barcodes than cells in Subpop5.*

Just like ED, CB2 also uses two sets of threshold for the initial 3-group classification. It is evident that these two thresholds have an impact over the performance of both ED and CB2. Similarly, the naive approach (single threshold) also uses thresholds. The paper did not provide sufficient evidence to support its intuition where there exists cell-enclosing droplets whose overall UMI counts are similar to empty droplets. Perhaps the naive approach has worse performance simply due to using non-optimal thresholds.

*Reviewer#1 also asked about this. We reiterate our response here. In short, there is no threshold at which the naive method works well. To illustrate this point, for SIMIA datasets we evaluated two simple thresholding approaches: T1 refers to identifying a barcode as a cell if the total count exceeds 50% of the inflection point and T2 is similar, but at 90% of the inflection point. As shown below, the T1 threshold does increase power in 7 of the 10 simulated datasets considered. However, in most of these datasets the increased power comes with greatly increased false discovery rates. In particular, at the T1 threshold, 9 of the 10 simulated datasets considered have FDR above the 1% target; 3 of the 10 have FDRs over 20%. Using the T2 threshold only increases power in 4 of the datasets and for 3 of these, the increase is less than 2%. However, as with the T1 threshold, the increased power comes with increased FDR. For T2, 3 of the 10 datasets have FDR that is not well controlled at the target 1%.*

*Figure: Average power (top panel) and average FDR (lower panel) from two thresholding methods (T1 and T2) and CB2 applied to SIMIA data (power and FDR are averaged over G1, G1.5, and G2 cells). In T1, barcodes are classified as cells if their total UMI count exceeds 50% of the inflection point. T2 is similar, but at 90% of the inflection point.*

We recommend the authors to provide further evidence to show cell-enclosing droplets could indeed have similar UMI counts to empty droplets.

*Below we show the distribution of UMI counts for background barcodes and cells identified uniquely by CB2 (left panel), identified in total by CB2 (middle panel), and identified by ED (right panel) for the Alzheimer dataset. As shown, the additional cells identified by CB2 are in the moderate count range [the 25th and 75th percentile of UMI counts for barcodes that are not background by default is 423-3873] and there is much overlap between background droplets and cell-enclosing droplets for a given UMI count.*

*Figure: Distribution of the number of cells and background barcodes identified by CB2 (left) and ED (right). The x-axis is log10 scaled ranging from lower threshold (100) to upper threshold (for the Alzheimer dataset, the upper threshold is 8136) since barcodes outside this range are not tested by either CB2 or ED.*

A evaluation over the impact of parameter choices between naive, ED and CB2 is also much desired.

*As shown in the figure on the previous page, altering the upper threshold leads to increased FDR for most simulated data sets. To investigate the impact of different lower thresholds, we evaluated results from SIMIA with the lower threshold set at 50 and at 150. As shown in the results below, there is little change in power or FDR and so both CB2 and ED seem to be robust to moderate changes in these parameters.*

*Figure: The average power and average false discovery rate (FDR) of CB2 and ED for SIM IA data (average taken over 5 simulated datasets) when the lower threshold is set to be 50 (left) and 150 (right). Since both CB2 and ED automatically identify high count barcodes as real cells (they are not subject to statistical test), we report results for all barcodes as well as those tested by CB2 and ED. The top panel shows the average power for tested barcodes; the middle panel for tested as well as high count barcodes. The bottom panel shows the average FDR. For the PanT4K dataset, all $ cells are above the upper threshold and so no barcodes were tested (as a result, power for tested barcodes is not defined).*

The rationale behind the simulation setup is not well explained in the manuscript. The authors simulate datasets by mixing the original dataset with multiple down-sized versions of itself at different scaling factors. This comment is also attached to the previous two comments. The simulation approach suggests that all cell types have equal chances of being under sampled in sequencing and become indistinguishable from the background noise. Without providing justifications that there does not exist a clear cut between cell-enclosing droplets and empty droplets in terms of total UMI counts and all cell types may get down sampled, it renders the simulation setup somewhat random and arbitrary.

*We considered two sets of simulations, both from the ED paper. The first (our SIM IA) exactly matches the simulations from the ED bioRxiv paper and the second (SIM IB) exactly matches their published paper. We did this for two reasons. The first is that the editors at Genome Biology request that any new method that improves upon a previously published approach be evaluated on identical simulated and case study data. The second is that the simulation seems realistic and reasonable to us. In particular, we think that sampling high count barcodes so that there “does not exist a clear cut between cell-enclosing droplets and empty droplets in terms of total UMI counts” is a strength of the simulation. If there was a clear difference between cell-enclosing droplets and empty droplets in terms of UMI counts (e.g. cell-enclosing droplets had uniformly larger counts than empty droplets), the simulation would seem artificially simple and we would expect all methods to work well.*

The significance of conducting statistical tests over an entire cluster instead of individual droplets is not clearly explained and, in our opinion, not sufficiently stressed.

*We regret that the advantages of testing on clusters (e.g. increased power) were not sufficiently stressed and have added further explanation and references to the methods section. In short, as you know, if individual features on test (here, barcodes) come from a common distribution, testing on combined features has increased power as the total number of tests is reduced and the signal to noise ratio is increased. We have added this important point and associated references to the third paragraph of the CB2 section in Methods. Specifically, that paragraph now reads:*

*“CB2 follows ED by filtering out genes with zero counts and dividing the remaining barcodes into three groups. However, instead of testing all barcodes from B_1 individually, CB2 first clusters barcodes and then tests tight clusters to identify those that differ from the background. As in methods for genome wide association studies (Mieth et al., 2016), gene co-expression network analysis (Botía et al., 2017), and de novo transcriptome analysis (Malik et al., 2018), clustering prior to testing increases power by reducing the total number of tests and increasing the signal to noise ratio. CB2 proceeds as follows:”*

Minor comments:

* Page 3 line 31-43. ED is poorly explained. "Similar to previous approaches" -> add citation.

*Done. Thank you.*

Does ED infers the upper and the lower threshold from the dataset?

*ED defaults to 100 for the lower threshold (but this can be change by a user); the upper threshold is taken to be the knee point, as described in their paper, and so we did not give details here.*

*"compromises power in many datasets" -> unclear. Perhaps "compromises its statistical power in identifying cells in many datasets"?*

*We have changed this sentence.*

*Page 4 line 19-29: hard to follow. Adding an overview of the simulation process would greatly help. Such as: simulation is conducted by combining three differently scaled datasets G1, G2 and G3.

*We have added an overview of the simulation process. The first paragraph of Results and*

*Discussion now reads “Briefly, given an input dataset, an inflection point dividing low from high count barcodes is determined. Low count barcodes are pooled to estimate the background distribution. Background barcodes are sampled from this distribution to match the total number and size of barcodes below the inflection point in the input dataset. Six thousand real cells are then generated as follows. First, two thousand barcodes are randomly sampled from the high count barcodes (referred to as G_1 cells); a second set of 2000 high count barcodes is sampled and then downsampled by 90% to give G_2 cells; the third set (G_1.5) is obtained by sampling 2000 barcodes from the high count range and downsampling by 50%. We note that in Lun et al., 2019, only G1 and G2 cells were considered. Here, G1.5 cells were added to better reflect real data.”*

*Page 4 line 48: "To further evaluate CB2, we applied CB2 and ED to the ten datasets evaluated in the simulation study". Is it not simulation? Is it a repeat of the previous paragraph or new results? Unclear.

*Good point. Thank you! We have changed this sentence to “To further evaluate CB2, we applied CB2 and ED to the ten case study datasets used to generate the simulated data as well as one additional dataset considered in the ED case study and compared the number of cells identified in common as well as those uniquely identified by each approach.”*

*The github has very limited information and example. A clear documentation with running examples should be provided so readers can follow and apply to their own datasets.

*Our package scCB2 has a well-documented vignette with runnable examples for each function. Details can be found at https://github.com/zijianni/scCB2. The source codes need to be downloaded and built to view the examples. Once the package is submitted to Bioconductor, the binary version will be available and a user will not need to build the package to view an HTML of the vignette. For your convenience, we have added an HTML of the vignette to the GitHub site.*

*References*

*Townes FW, Hicks SC, Aryee MJ, Irizarry RA. Feature selection and dimension reduction for single-cell RNA-Seq based on a multinomial model. Genome Biol. 2019;20:295.*

*Mathys H, Davila-Velderrain J, Peng Z, Gao F, Mohammadi S, Young JZ, et al. Single-cell transcriptomic analysis of Alzheimer’s disease. Nature. 2019;570:332–7.*

*Mieth B, Kloft M, Rodríguez JA, Sonnenburg S, Vobruba R, Morcillo-Suárez C, et al. Combining multiple hypothesis testing with machine learning increases the statistical power of genome-wide association studies. Sci Rep. 2016;6:1–14.*

*Botía JA, Vandrovcova J, Forabosco P, Guelfi S, D’Sa K, Hardy J, et al. An additional k-means clustering step improves the biological features of WGCNA gene co-expression networks. BMC Syst Biol. 2017;11:47.*

*Malik L, Almodaresi F, Patro R. Grouper: graph-based clustering and annotation for improved de novo transcriptome analysis. Bioinformatics. 2018;34:3265–72.*

*Lun ATL, Riesenfeld S, Andrews T, Dao TP, Gomes T, Marioni JC. EmptyDrops: distinguishing cells from empty droplets in droplet-based single-cell RNA sequencing data. Genome Biol. 2019;20:63.*

**Second round of review**

**Reviewer 1**

The authors have sufficiently addressed the comments brought up in my first review.

**Reviewer 2**

We appreciate the clarifications made in the revision and we believe the authors have address most of our technical concerns. We have no doubt that the reasoning of CB2 is sound and that the manuscript has clearly shown that CB2 is able to recover previously overlooked cells. We understand and agree that 1) testing a group of transcriptomically similar cells yields greater statistical power than testing cells individually;

and 2) the idea of testing in groups is orthogonal to whether certain cell types are systematically down-sampled during single cell sequencing.

However, clipping dead cells/empty droplets is not the only reason that low-RNA-count cells are excluded from downstream analysis. Cells of low RNA capture rates are more prune to high variations stem from low sampling rate and are more likely to create noise and artifacts in downstream analysis. The low-RNA-content cells rescued by CB2 are likely to be designated as low-quality cells in downstream analysis and are again subject to removal in order to maintain a small dynamic range in cell UMI counts to avoid false positives.

We want to re-iterate the comments are not related to the technical soundness of CB2 in discovering low-RNA-content cells but rather questions the importance of doing so. We believe the significance of CB2 could be greatly improved if proven its effectiveness in such scenarios by showing additional real example in addition to Alzheimer’s disease dataset. For instance, in cancer research, researchers could be interested in tumor infiltrating lymphocytes (TIL) but due to hyperexpression of cancer cells and T cell exhaustion, TILs might have relatively low RNA content and could be undesirably excluded from the cell population in current analytical settings.

Minor:

1. Did the authors try different clustering methods other than hierarchical clustering for the clustering step? Is it sensitive to the type of clustering method used.

2. For Figure 2(a), it is hard to distinguish between pink and dark pink colors.

3. What is the computational speed for scCB2? Is there any benchmark performance for scCB2?

4. DE analysis (supplementary Figure 7): Including more cells in each group may increase the power (lower the pvalue) but is unnecessary to increase the folder change. Are newly included cells evenly distributed between cases and controls? Brief discussion is needed.

**Authors Response**

**Point-by-point responses to the reviewers’ comments:**

Reviewer #2: We appreciate the clarifications made in the revision and we believe the authors have address most of our technical concerns. We have no doubt that the reasoning of CB2 is sound and that the manuscript has clearly shown that CB2 is able to recover previously overlooked cells. We understand and agree that 1) testing a group of transcriptomically similar cells yields greater statistical power than testing cells individually; and 2) the idea of testing in groups is orthogonal to whether certain cell types are systematically down-sampled during single cell sequencing.

However, clipping dead cells/empty droplets is not the only reason that low-RNA-count cells are excluded from downstream analysis. Cells of low RNA capture rates are more prune to high variations stem from low sampling rate and are more likely to create noise and artifacts in downstream analysis. The low-RNA-content cells rescued by CB2 are likely to be designated as low-quality cells in downstream analysis and are again subject to removal in order to maintain a small dynamic range in cell UMI counts to avoid false positives.

*We are not sure what is meant exactly by this comment, but it seems there is some concern about the amount of low-count cells identified by CB2 and the quality of these cells in downstream analysis. Toward this end, we note the following points.*

*1.	Most CB2 identifications are not in the low count range. Below we show results from the Alzheimer’s data (these results were shown in our initial response but are also shown below for convenience). We also show results from the three other case study datasets analyzed in the paper. Specifically, Figures 1-4 below show distributions of the total counts of cells and background barcodes identified uniquely by CB2, identified in total by CB2, and identified by ED. In particular, for the Alzheimer’s (PBMC8K, mbrain1K, and placenta) data, 59% (70%, 48%, and 16%) of the unique CB2 identifications are above the 25th percentile of total counts across all the cells identified.*

*2.	CB2’s false positive rate is well controlled in simulation studies (for both high count and low count cells) which further supports the claim that the barcodes that CB2 identifies are, in fact, true cells.*

*3.	Most of the novel CB2 identifications add cells to existing subpopulations. The gene expression profiles of these “extra” cells is very similar to other cells in the subpopulation, and they differ from the background. This is shown in Figure 2(b) and 2(c) for the Alzheimer’s dataset and in panels(b) and (c) of Supplementary Figures 8, 9, and 10 for the PBMC8K, mbrain1K, and placenta datasets, respectively. In addition, marker gene expression analyses in these datasets also strongly suggest that the extra cells identified by CB2 are not false positives (panels(d) of Figure 2 and Supplementary Figures 8-10).*

*4.	In Figure 3 (new), we consider the PBMC8K data which is well characterized by specific markers defining specific subpopulations. We show that marker gene expression in the CB2 unique cells is similar to marker gene expression in cells identified by both ED and CB2, even in the relatively low count subpopulations.*

*5.	As noted in Lun et al. , 2019, any method for distinguishing cells from background barcodes is technically correct in identifying low-quality cells (which are often low count) given that damaged cells exhibit expression profiles that differ from the background. Specifically, mitochondrial gene expression is often high in damaged cells. Such cells are typically not of interest in downstream analysis and it is therefore recommended by both ED and CB2 that they be removed (the GetCellMat function in R/scCB2 is provided for this purpose).*

*Taken together, analyses of both case study and simulated data provide considerable evidence that CB2 identifies additional cells (both low and relatively high count) that lead to improved downstream analyses.*

*Figure 1: Distribution of the number of cells and background barcodes identified uniquely by CB2 (left), identified in total by CB2 (middle), and identified by ED (right) for the Alzheimer’s case study dataset. The x-axis is log10 scaled ranging from lower threshold (100) to upper threshold (for this Alzheimer dataset, the upper threshold is 8136) since barcodes outside this range are not tested by either CB2 or ED. The barcodes identified by CB2 are not concentrated in the low count range. Specifically, 59% of the unique CB2 identifications are above the 25th percentile of total counts across all the cells identified.*

*Figure 2: Distribution of the number of cells and background barcodes identified uniquely by CB2 (left), identified in total by CB2 (middle), and identified by ED (right) for the PBMC8K dataset. The x-axis is log10 scaled ranging from lower threshold (100) to upper threshold (for this PBMC8 dataset, the upper threshold is 3595) since barcodes outside this range are not tested by either CB2 or ED. The barcodes identified by CB2 are not concentrated in the low count range. Specifically, 70% of the unique CB2 identifications are above the 25th percentile of total counts across all the cells identified.*

*Figure 3: Distribution of the number of cells and background barcodes identified uniquely by CB2 (left), identified in total by CB2 (middle), and identified by ED (right) for the mbrain1K dataset. The x-axis is log10 scaled ranging from lower threshold (100) to upper threshold (for this mbrain1K dataset, the upper threshold is 7185) since barcodes outside this range are not tested by either CB2 or ED. The barcodes identified by CB2 are not concentrated in the low count range. Specifically, 48% of the unique CB2 identifications are above the 25th percentile of total counts across all the cells identified.*

*Figure 4: Distribution of the number of cells and background barcodes identified uniquely by CB2 (left), identified in total by CB2 (middle), and identified by ED (right) for the placenta dataset. The x-axis is log10 scaled ranging from lower threshold (100) to upper threshold (for this placenta dataset, the upper threshold is 5043) since barcodes outside this range are not tested by either CB2 or ED. The barcodes identified by CB2 are not all in the low count range. Specifically, 16% of the unique CB2 identifications are above the 25th percentile of total counts across all the cells identified.*

We want to re-iterate the comments are not related to the technical soundness of CB2 in discovering low-RNA-content cells but rather questions the importance of doing so. We believe the significance of CB2 could be greatly improved if proven its effectiveness in such scenarios by showing additional real example in addition to Alzheimer's disease dataset. For instance, in cancer research, researchers could be interested in tumor infiltrating lymphocytes (TIL) but due to hyperexpression of cancer cells and T cell exhaustion, TILs might have relatively low RNA content and could be undesirably excluded from the cell population in current analytical settings.

*In addition to results from the Alzheimer’s data, we had included results from three other datasets in the supplement (PBMC8K, mbrain1K and placenta). However, we did not discuss these results in detail, and we did not mention specific results in the manuscript. In this revised version, we have added 3 paragraphs and a new figure (Figure 3) to the Results section which expand upon results from the Alzherimer’s data and include detailed results from the PBMC8K data. The additions are highlighted in the revised manuscript, as requested, and also shown below for convenience.*

*As an example, Figure 2 and Supplementary Figure 6 show results from the Alzheimer data[5] where CB2 identifies 18% more cells. A detailed look at the unique CB2 identifications suggests that the extra cells identified are not false positives, but rather they add to existing excitatory neuron and inhibitory neuron sub-populations, and also reveal a novel subpopulation consisting of 209 cells. Specifically, Figure 2(b) and Figure2(c) show distribution plots and an expression heatmap of the 100 genes having the highest average expression in Subpop1 (the largest subpopulation) for cells identified by both CB2 and ED as well as those identified uniquely by CB2. As shown, cells uniquely identified by CB2 have a distribution similar to other cells, and they differ from the background. Using the marker genes from Mathys et al. 2019[5], Figure 2(d) and Supplementary Figure 6(b) suggest that cells identified uniquely by CB2 in Subpops1-4 are neurons, as they show relatively high expression of neuron marker genes SYT1, SNAP25, and GRIN1. More specifically, the CB2 cells in Subpops 1-2 exhibit high expression of excitatory neuronal markers whereas the cells in Subpops 3-4 appear to be inhibitory neurons (Supplementary Figure 6(c) and (d)). The novel subpopulation (Subpop5) uniquely shows high expression of both oligodendrocyte and astrocyte marker genes, suggesting that this group may be mixed phenotype glial cells[6] (Supplementary Figure 6 (e) and (f)).*

*By increasing the number of real cells identified, CB2 also improves the power to differentiate Alzheimer’s patients from controls. Specifically, Mathys et al., 2019[5] profiled expression from the prefrontal cortex of 24 patients showing Alzheimer’s disease pathology (AD) as well as 24 age-matched controls, and they validated differentially expressed genes in different cell types, including 9 genes in excitatory neurons and 9 in inhibitory neurons. Supplementary Figure 7 shows that by identifying additional cells, CB2 improves downstream differential expression analysis by resulting in more significant p-values and stronger fold changes.*

*In a second case study (PBMC8K), CB2 increases the number of cells identified across six subpopulations by over 80% (Supplementary Table 1). Results are shown in Figure 3 and Supplementary Figure 8. Similar to the Alzheimer’s data analysis, Supplementary Figures 8(b) and 8(c) show that cells identified uniquely by CB2 in Subpop1 (the largest subpopulation) have an expression profile that is similar to other cells, and differs from the background. Figure 3 provides a detailed look at marker gene expression for the well characterized PBMC8K cells using markers considered in Zheng et al ., 2017[2]. As shown in Figure 3(b), CB2 identifies additional CD14+ Monocytes, T-cells, B-cells, and megakaryocytes. Results from two additional datasets are shown in Supplementary Figures 9-10.*

*Figure 3: Results from the PBMC8K dataset. (a) t-SNE plot of cells identified by CB2 and ED. High-count barcodes exceeding an upper threshold are identified as real cells by both methods without a statistical test (dark pink); barcodes identified as cells by both methods following statistical test are shown in pink. Cells identified uniquely by CB2 (yellow) and ED (black) are also shown. CB2 increases the number of cells identified across the six subpopulations by over 80% (Supplementary Table 1). (b) Subpopulations 1-5 ordered by median normalized UMI count along with marker gene expression for each subpopulation. Marker gene expression in cells uniquely identified by CB2 is similar to that in other groups, and differs from the background. Subpopulation 5 contained no high count common cells; subpopulation 6 contained no unique CB2 identifications and is therefore not shown in panel (b).*

Minor:

1.	Did the authors try different clustering methods other than hierarchical clustering for

the clustering step? Is it sensitive to the type of clustering method used.

*We expect that most clustering algorithms will work similarly for our purposes since there are minimal differences between standard general-purpose clustering methods*

*( https://www.ncbi.nlm.nih.gov/pmc/articles/PMC6134335/ ). We found this to be the case when we considered k-means clustering instead of hierarchical clustering. We implemented hierarchical clustering in the code because it can be applied to any distance metric. CB2 uses Pearson correlation as the distance metric as it is consistent with the correlation-based test statistic and makes fewer assumptions*

*( https://www.sciencedirect.com/science/article/pii/S0098299717300493 ). Finally, it should be noted that CB2 evaluates clusters and filters out poor clusters if necessary. This ensures that barcodes coming from the same subpopulation are correctly clustered together and can be tested together to improve power.*

2.	For Figure 2(a), it is hard to distinguish between pink and dark pink colors.

*Yes, these colors were chosen since both sets of cells are identified in common by CB2 and ED. The only difference between the sets of cells is that the lighter pink cells were identified by both CB2 and ED after being tested whereas the darker pink cells were identified by both CB2 and ED because they were high count (no statistical test required). This distinction is very minor, and that is why we chose very minor differences in the colors.*

3.	What is the computational speed for scCB2? Is there any benchmark performance for scCB2?

*Thank you. The computational speed is related to the size and structure of input datasets. As a reference, scCB2 running on a medium-size dataset (around 30,000 genes and 8,000 cells after cell calling) using 6 cores takes less than 10 minutes. We have added a note on this to our vignette.*

4.	DE analysis (supplementary Figure 7): Including more cells in each group may increase the power (lower the pvalue) but is unnecessary to increase the folder change. Are newly included cells evenly distributed between cases and controls? Brief discussion is needed.

*Thank you for this point. We agree that including more cells need not alter the fold change particularly if the new cells have the same profiles as existing cells. An interesting fact for this analysis is that some of the extra cells are expressing stronger cell-type-specific marker genes. For example, in Supplementary Figures 6(b)-(e), we show that marker genes typically have higher expression in CB2 unique cells than in common cells which further validates that CB2 is finding real cells with stronger biological signals. We have added this important point to the legend of Supplementary Figure 7.*

*And the proportion of novel cells is roughly similar between cases and controls. Specifically, among the 80110 cells identified by CB2, 39479 (49.3%) come from AD-pathology patients. With ED, among the 69471 cells identified, 32755 (47.1%) come from AD-pathology patients.*

**Third round of review**

**Reviewer 2**

Authors have addressed all of my comments.
